# INTRINSIC CAPACITY OF OLDER ADULTS IN SINGAPORE USING WHO INTEGRATED CARE FOR OLDER PEOPLE (ICOPE) FRAMEWORK

**DOI:** 10.1093/geroni/igad104.2875

**Published:** 2023-12-21

**Authors:** Carol Ma, Angela Y M Leung, Denise Chua, Wai Choo Teo, Laura Bee Gek Tay, Wai Chong Ng

**Affiliations:** Singapore University of Social Sciences, Singapore, Singapore; Hong Kong Polytechnic University, Hong Kong, Hong Kong; Singapore University of Social Sciences, Singapore, Singapore; Singapore University of Social Sciences, Singapore, Singapore; Sengkang General Hospital, Singapore, Singapore; NWC Longevity Practice, Singapore, Singapore

## Abstract

The World Health Organization (WHO) proposed the Integrated Care for Older People (ICOPE) approach to guide health systems in better supporting the intrinsic capacity and functional ability of older adults to enable healthy aging (Briggs et al., 2018; de Carvalho et al., 2019). This approach is aligned with Singapore’s Healthier SG initiative, which aims to build a good healthcare system that promotes better health and quality of life for everyone. Recent studies have shown that older adults experience intrinsic capacity decline in various countries, including Singapore, highlighting the importance of targeted interventions to maintain functionality and quality of life in old age (Beard et al., 2019; Liu et al., 2021; Tay et al., 2022). In this cross-sectional study, 367 participants were assessed by 43 ICOPE assessors, of whom 77.4% (n=284) had impairments in intrinsic capacity. The three most prevalent intrinsic capacity impairments were visual impairment (42%), hearing loss (33.5%), and cognitive decline (31.3%), followed by limited mobility (24.3%), malnutrition (16.1%), and depressive symptoms (16.1%). Furthermore, 22.6% of participants were unaware of any elderly care services available in the community, and 8.2% did not know where to seek help in case of problems. These findings emphasize the need for ICOPE assessments at the community level to promote early detection of intrinsic capacity impairments and interventions. More discussion of the care pathway, together with health professionals, older adults, and caregivers, should be the next step to offer diagnostic assessment and guide self-management of intrinsic capacity impairment for among older adults.

